# Changes in speckle-tracking-derived mechanical dispersion index are associated with 30-day readmissions in acute heart failure

**DOI:** 10.1186/s13089-019-0125-8

**Published:** 2019-05-02

**Authors:** Mark Favot, Robert Ehrman, Laura Gowland, Ashley Sullivan, Brian Reed, Aiden Abidov, Phillip Levy

**Affiliations:** 1 Department of Emergency Medicine, Wayne State University School of Medicine, 6071 W. Outer Dr., Lourdes 447-D, Detroit, MI 48235 USA; 2 Department of Internal Medicine, Division of Cardiology, Wayne State University School of Medicine, Detroit, MI USA; 3 Cardiovascular Research Institute, Wayne State University School of Medicine, Detroit, MI USA

**Keywords:** Longitudinal strain, Speckle-tracking, Mechanical dispersion index, Readmissions, Echocardiography, Point-of-care echocardiography

## Abstract

**Background:**

The objective of the present study was to evaluate the relationship between speckle-tracking-derived parameters left ventricular (LV) mechanical dispersion index (MDI), defined as the standard deviation of the time-to-peak longitudinal strain of all segments analyzed of the LV, and global longitudinal strain (GLS) and 30-day post-discharge outcomes (death and readmission to the hospital) in patients with acute heart failure (AHF).

**Methods:**

We performed a prospective observational study of selected emergency department patients with a primary diagnosis of AHF. Point-of-care echocardiograms were performed at baseline (prior to, or concurrent with the initiation of treatment) and 23 h post-enrollment. Offline speckle-tracking analysis was utilized to calculate GLS and MDI. The primary outcome was 30-day readmissions.

**Results:**

A total of 31 patients were included, 13 of whom were readmitted within 30 days. Patients who were not readmitted to the hospital experienced an average relative improvement in MDI of 24% from baseline to 23 h (84 ms to 64 ms), while patients who were readmitted experienced an average relative worsening in MDI of 6% (66 ms to 70 ms) from baseline to 23 h.

**Conclusions:**

MDI has promise as a treatment response variable in admitted patients with AHF; however, further study is needed.

## Introduction

Once a diagnosis of acute heart failure (AHF) has been established, conventional 2D and Doppler echocardiography techniques provide limited relevant information to guide treatment decisions at the point-of-care (POC) [1]. Left ventricular ejection fraction (LVEF) is the most extensively investigated echocardiographic parameter, but it does not appreciably change during the acute treatment phase of AHF [2] and it provides limited actionable information when it comes to the selection of specific therapies that may be most suitable for a given patient with AHF [3, 4].

LV strain utilizing speckle-tracking technology is a relatively new quantitative method for evaluating LV systolic function, and while it has been well studied in chronic HF [5–9], to date, there has been only one published report prospectively investigating its use, pre-treatment, in AHF [10]. Although there are numerous ways to measure LV strain utilizing speckle-tracking, global longitudinal peak systolic strain (GLS) is the most well studied [11]. GLS represents a summative measure of the degree of longitudinal shortening of each of the segments of the LV (typically 16–18 depending on the vendor and software being utilized) that are analyzed. GLS has been found to be associated with AHF readmission, independent of and incremental to clinical and basic echocardiographic parameters [12].

LV mechanical dispersion index (MDI) is the standard deviation (SD) of the time-to-peak longitudinal strain in each of the segments of the LV that are analyzed [13] and provides unique insight into mechanical dyssynchrony. While LV MDI has previously been shown to be a predictor of ventricular arrhythmias post-myocardial infarction, a predictor of adverse events following cardiac resynchronization therapy (CRT), and a discriminator of hypertrophic cardiomyopathy versus athlete’s heart [13–15], it has yet to be considered as a potential measure of treatment effectiveness in AHF. A recent investigation by Chan et al. [16] found LV MDI to be an independent predictor of outcome in AHF patients after adjustment for baseline variables. The objective of the present study was to evaluate the relationship between the speckle-tracking-derived parameters LV MDI and GLS obtained pre- and 23-h post-treatment, and 30-day post-discharge outcomes (death and readmission to the hospital) in patients with AHF.

## Materials and methods

### Setting

This prospective observational study was conducted in the emergency department (ED) at 2 large, urban, tertiary-care centers serving approximately 80,000 and 100,000 patients per year, a majority of whom (> 90%) are African American. The study received approval by the Institutional Review Board (IRB).

### Selection of participants

From February, 2015 to July, 2017 a convenience sample of eligible ED patients with a primary diagnosis of AHF treated with intravenous (IV) therapy were recruited during hours, where a study team member was available to perform a POC echocardiogram. Whenever possible, we attempted to enroll patients prior to administration treatment. The inclusion criteria were: 18 years of age or greater, primary admitting diagnosis of AHF (determined by the attending emergency physician) being treated with IV therapy, and the ability to provide informed written consent. Exclusion criteria were: the lack of immediate availability of a study team member capable of performing the echocardiograms, the need for emergent, resuscitative intervention (e.g., cardiopulmonary resuscitation, endotracheal intubation, and cardioversion), rapid atrial fibrillation/flutter or any other tachyarrhythmia requiring rate or rhythm control, heart rate persistently greater than 120 beats/min, poor image quality precluding speckle-tracking analysis, plans for emergent percutaneous coronary intervention (PCI) from the ED, pregnancy, incarceration, and plans for to another institution.

Potentially eligible subjects were identified by trained research assistants after discussion with the treating physician to enquire about the primary diagnosis and treatment plan so that all efforts could be made to enroll the patient, obtain consent and perform the baseline POC echocardiogram prior to the initiation of therapy. We allowed for a 20-min window from the initiation of therapy until the baseline POC echocardiogram—if images were unable to be obtained within this window, the patient was not eligible for enrollment.

### Study design

**Fig. 1:**
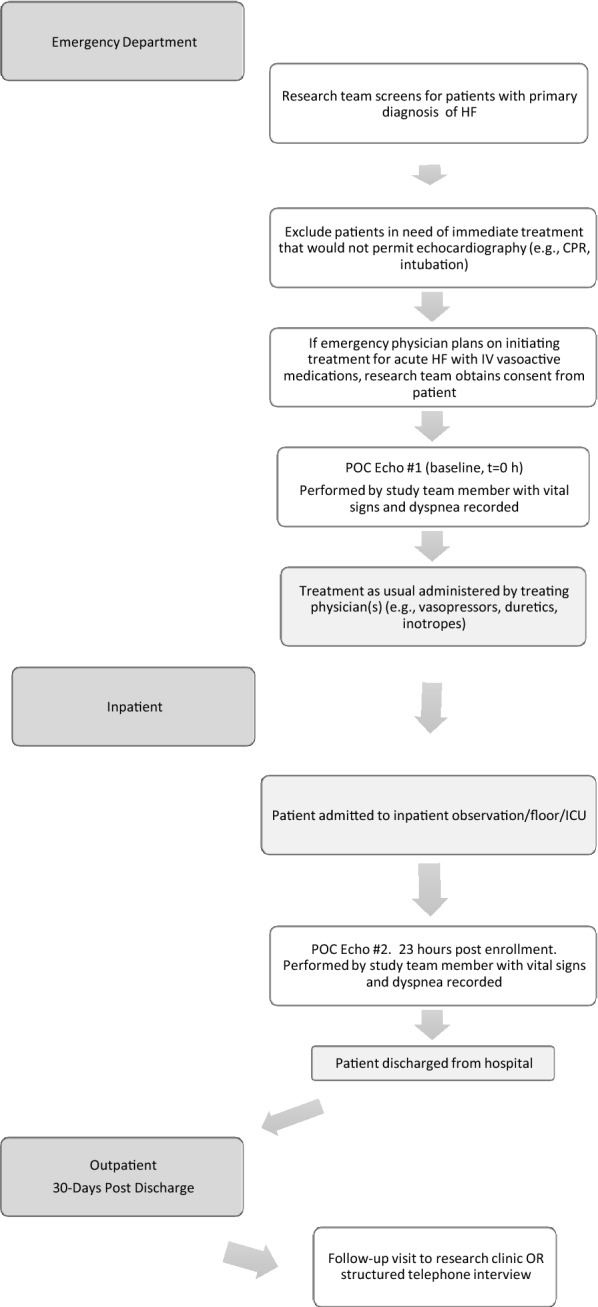
Flow diagram for the study from enrollment in the emergency department to inpatient follow-up echocardiogram to outpatient follow-up at 30-day post-discharge

At the time of enrollment, study subjects underwent a baseline POC echocardiogram that was performed by a study team member consisting of 2 ultrasound fellowship-trained emergency physicians with expertise in POC echocardiography and speckle-tracking, as well as a certified cardiac sonographer who is an employee of our department’s division of clinical research. Vital signs and therapeutic interventions were recorded at the time of the baseline echocardiogram. Baseline demographic data, comorbidities, and home medications were also recorded at this time. Following the 1st echocardiogram, patients underwent usual care for AHF; a 2nd POC echocardiogram was performed 23 h following enrollment. Vital signs, laboratory findings, and further therapies and interventions up to this point were also recorded. Following the 2nd POC echocardiogram, subjects were tracked in-hospital until discharge at which time arrangements were made for a 30-day follow-up telephone interview to ascertain post-discharge outcomes (Fig. 1).

### Point-of-care echocardiograms

All POC echocardiograms were performed using the same protocol with a portable ultrasound system (*Vivid q* by *GE*, Milwaukee, WI). Study echocardiograms were limited and included only the apical imaging window utilizing the 4-chamber, 2-chamber, and long axis views. After the recording of these ultrasound clips, the POC echocardiogram was considered complete and stored both in the ultrasound system and transmitted to our EchoPAC (*GE* Milwaukee, WI, USA) workstation for subsequent offline analysis by an investigator who was blinded to treatment, all clinical and laboratory variables and outcome.

### Strain analysis

**Fig. 2:**
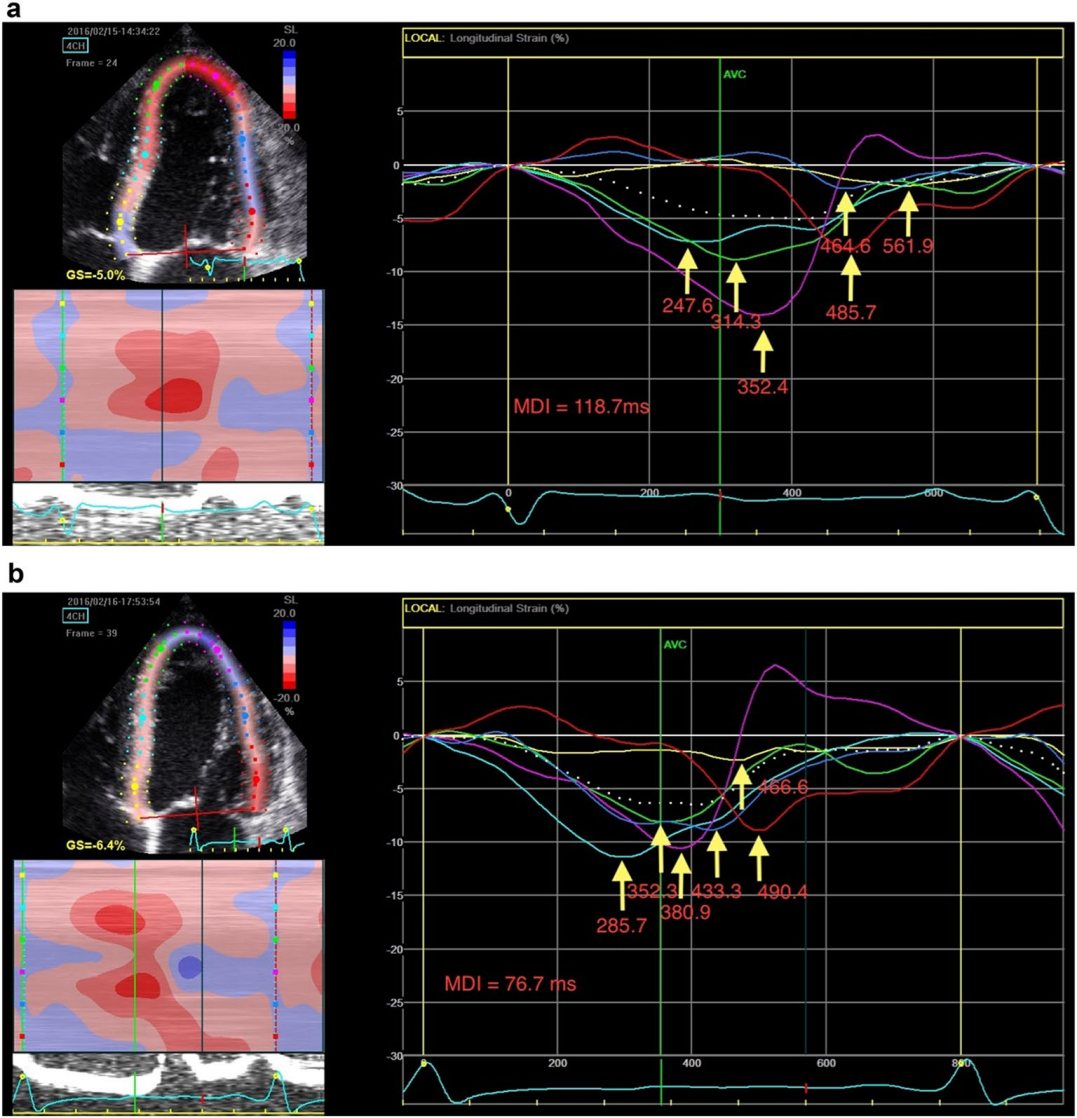
Strain tracings from an apical 4 chamber view of a patient with acute heart failure at: **a** baseline and **b** 23-h follow-up. Each yellow arrowhead points to the peak longitudinal strain for one of the 6 segments of the left ventricular wall visualized in this view. The values adjacent to each arrowhead represent the time-to-peak longitudinal strain for the corresponding segment. Mechanical dispersion index (MDI) is calculated by calculating the standard deviation for the time-to-peak longitudinal strain values. After 23 h of treatment, the MDI has improved (decreased) by approximately 35% in this patient who had a favorable post-discharge outcome

Offline analysis was conducted according to the recommendation of the American Society of Echocardiography and the European Society of Cardiovascular Imaging using commercially available software (EchoPAC version 110.1.1 BT 11, *GE* Milwaukee, WI, USA). We calculated GLS from the baseline POC echocardiogram and again from the 2nd POC echocardiogram performed 23 h following enrollment using the embedded Automated Function Imaging (AFI) tool. Using the longitudinal strain tracings (Fig. 2), LV MDI was subsequently calculated for baseline and repeat POC echocardiograms by calculating standard deviation of the time-to-peak longitudinal (negative) strain for each of the segments analyzed. All of the strain analyses were performed by a single operator (MF) who was blinded to all of the clinical, radiological and laboratory data at the time of analysis. The automated tracings provided by the AFI software were utilized without any manual adjustments unless it was noted by the operator that the automatic tracings of the endocardial border were grossly inappropriate. Only echocardiograms with frame rates between 40 and 80 frames per second were included for analysis. Echocardiograms that had more than 1 non-trackable segment in a single imaging plane were excluded from analysis as GLS is unable to be calculated when any of the imaging planes are missing > 1 segmental strain. Our prior study demonstrated a very high inter-rater reliability between sonographers of with a concordance correlation coefficient of 0.993 [10].

### Primary outcomes and data analysis

All statistical analyses were performed using SAS 9.4. Categorical variables were compared using Chi squared, or Fisher’s exact test in cases were expected cell counts was insufficient, and continuous measures were compared using the paired *T* test and Wilcoxon Rank sum (both approaches were consistent in categorizing results at the 0.05 significance level). Logistic regression was performed to investigate the relationship between GLS, MDI, and our primary outcome which was readmission or death within 30 days of discharge. We assessed initial, final, and percent change to investigate whether absolute measure, or directional trends in MDI and GLS were useful predictors of patient outcome. Single and multiple logistic regressions were performed using measures of MDI and GLS individually, and in combination. The c-statistic [area under receiver–operator characteristic curve (AUC)] was reported as a measure of logistic regression performance, and Wald’s Chi square was used to evaluate predictor significance in each model.

## Results

### Characteristics of study subjects

A total of 44 patients were enrolled, 13 were ultimately excluded from the final analysis because of technical issues that rendered one of their echocardiograms unsuitable for strain analysis (7 due to poor ECG tracing, 5 due to poor endocardial border definition, and 1 patient with a heart rate > 120 beats/min on the 23-h echocardiogram). This left a final cohort of 31 patients (all African–American), 16 of whom were male (51.6%) with a mean age of 56.6 years (SD = 11.5 years). All 31 patients had abnormally elevated NT-proBNP concentrations and chest radiograph findings compatible with AHF. Mean GLS at baseline for all patients was − 6.5% (SD = 3.3%) and the mean GLS at 23 h follow-up for all patients improved to − 7.6% (SD = 3.4%). Mean MDI at baseline for the entire cohort was 76 ms (SD = 29 ms), while the mean MDI at 23 h follow-up for all patients had improved to 66 ms (SD = 16 ms). Thirteen (41.9%) patients were readmitted within 30 days and none died.

Data for the study cohort are presented in Table 1. To evaluate the representativeness of the cohort, we included data for a sample of 235 AHF patients from our institution who were enrolled in an on-going, ED-based AHF registry between March 2016 and August 2017. Overall the convenience sample that we enrolled for the present study was similar to this large registry with a high prevalence of elevated blood pressure, underlying hypertension and diabetes mellitus.

**Table 1:** Clinical and echocardiographic characteristics of study subjects

Parameter	30-day outcome		*p* value	Comparison EMROC cohort (n = 235)
	Favorable (*n* = 18)	Readmission (*n* = 13)		
Age, mean in years	57.2	55.9	0.77	58.5
Gender, female *n* (%)	7 (38.9%)	8 (61.5%)	0.29	116 (49.4%)
African American, *n* (%)	18 (100%)	13 (100%)	1.0	218 (92.8%)
DM, *n* (%)	7 (38.9%)	4 (30.8%)	0.72	100 (42.6%)
CAD, *n* (%)	9 (50.0%)	10 (76.9%)	0.16	
HTN, *n* (%)	16 (88.9%)	11 (84.6%)	1.0	224 (95.3%)
CKD, *n* (%)	5 (27.8%)	2 (15.4%)	0.67	76 (32.3%)
COPD, *n* (%)	7 (38.9%)	5 (38.5%)	1.0	109 (46.6%)
Systolic BP at baseline, mean	165.3 mm Hg	172.9 mm Hg	0.51	160.0 mm Hg
NIPPV support	4 (22.2%)	2 (15.4%)	0.63	10 (4.3%)
NT-proBNP, median	6787 pg/mL (IQR 3353–9077)	6022 pg/mL (IQR 2179–10,434)	0.61	3345 pg/mL (IQR 1335–8413)
Any troponin in first 23 h > 99th percentile limit, *n* (%)	10 (55.6%)	8 (61.5%)	0.74	140 (59.6%)
Creatinine, mean	1.88 mg/dL	1.68 mg/dL	0.78	1.2 mg/dL
Vasodilator therapy, *n* (%)	5 (27.8%)	6 (46.2%)	0.45	77 (32.8%)
Inotropic therapy, *n* (%)	0 (0%)	0 (0%)	1.0	1 (0.4%)
Diuretic dosage in first 23 h, mean	63.3 mg	67.7 mg	0.75	42.6 mg
Echocardiographic data				
LVEF (%), mean (95% CI)	32 (26, 41)	33 (26, 41)	0.98	43%
Baseline GLS (%), mean (95% CI)	− 5.7 (− 7.0, − 4.3)	− 7.7 (− 10.0, − 5.4)	0.09	N/A
23-h GLS (%), mean (95% CI)	− 7.1 (− 8.6, − 5.7)	− 8.2 (− 10.6, − 5.7)	0.42	N/A
Absolute change GLS (%), mean (95% CI)	− 1.48 (− 2.5, − 0.43)	− 0.45 (− 1.49, 0.59)	0.09	N/A
Percentage change GLS (%), mean (95% CI)	− 61.9 (− 126, 2.3)	− 8.1 (− 27.3, 11.1)	0.08	N/A
Baseline MDI (ms), mean (95% CI)	84 (67, 101)	66 (55, 76)	0.07	N/A
23-h MDI (ms), mean (95% CI)	64 (57, 70)	70 (59, 81)	0.26	N/A
Absolute change MDI (ms), mean (95% CI)	− 20 (− 34.6, − 5.4)	4.3 (− 6.8, 15.3)	0.007	N/A
Percentage change MDI (%), mean (95% CI)	− 16.7 (− 28.8, − 4.7)	11.0 (− 6.8, 28.8)	0.008	N/A

*EMROC* Emergency Medicine Research and Outcomes Consortium, *DM* diabetes mellitus, *CAD* coronary artery disease, *HTN* hypertension, *CKD* chronic kidney disease, *COPD* chronic obstructive pulmonary disease, *BP* blood pressure, *NIPPV* non-invasive positive pressure ventilation, *LVEF* left ventricular ejection fraction, *GLS* global longitudinal strain, *MDI* mechanical dispersion index

### Main results

As shown in Table 1, with the exception of findings on strain imaging, there were no significant group differences between subjects that had a favorable post-discharge outcome and those who were readmitted. Regarding the strain findings, among patients who were not readmitted, the baseline GLS was − 5.7% (SD = 2.7%), while the 23 h follow-up GLS had improved to − 7.1% (SD = 2.9%) with an average absolute improvement in GLS over 23 h of 26%. For patients that were readmitted within 30 days, the mean baseline GLS was − 7.7% (SD = 3.8%), while the mean GLS at 23 h follow-up was –8.2% (SD = 4.0%) resulting in an average absolute improvement in GLS over 23 h of 6%. For the patients with a favorable post-discharge outcome, the mean MDI at baseline was 84 ms (SD = 34 ms), with improvement to 64 ms (SD = 13 ms) at 23 h follow-up. This resulted in an average relative improvement in MDI of 24%. In patients readmitted within 30 days, the mean MDI at baseline was 66 ms (SD = 18 ms) and the mean MDI at 23 h follow-up was 70 ms (SD = 19 ms). There was an average relative worsening in MDI for the readmitted patients over 23 h of 6%.

**Fig. 3:**
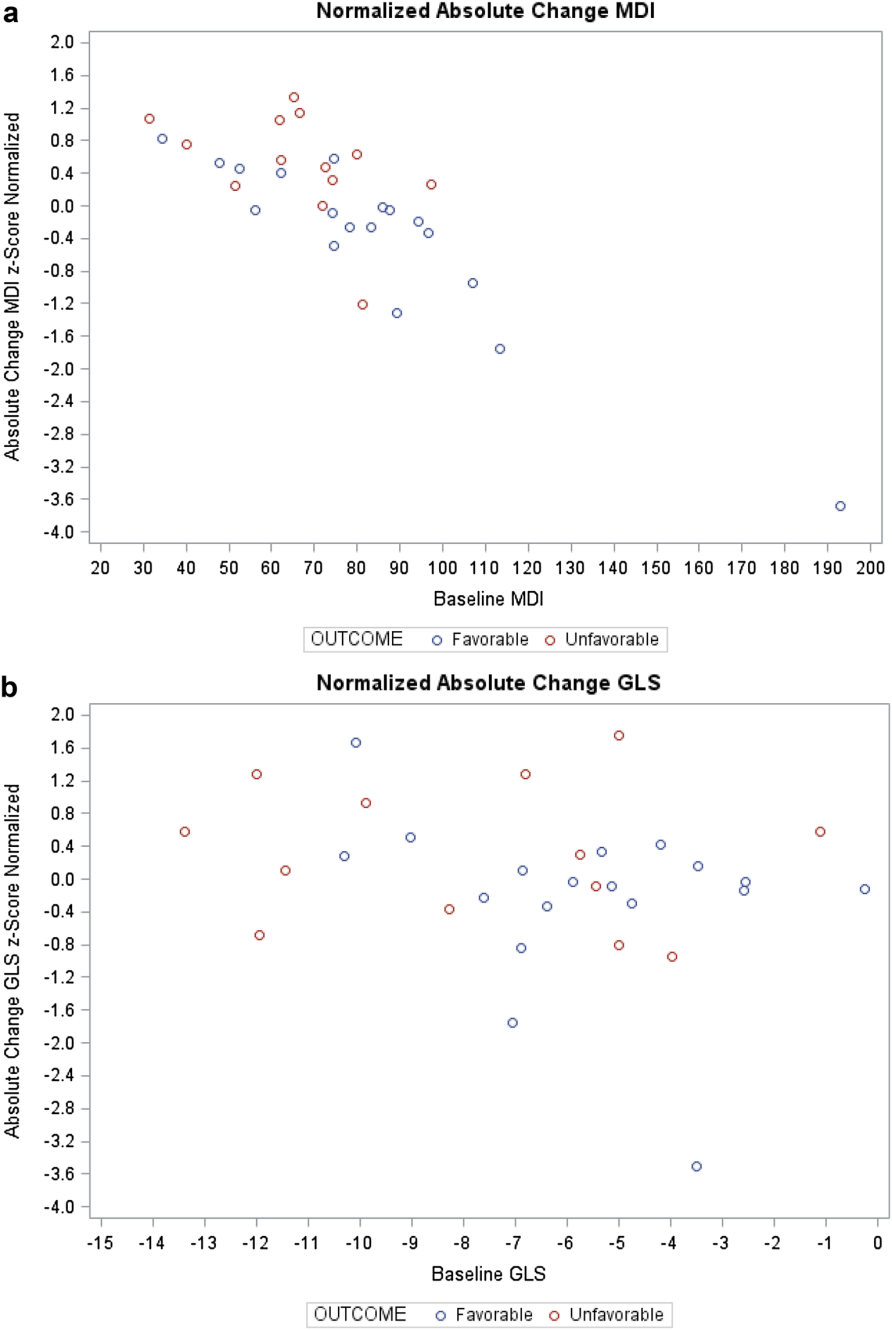
**a** Scatter plot of baseline mechanical dispersion index (MDI) vs. z-score normalized change in MDI (Absolute). There is a fairly clear delineation between patients with favorable vs. unfavorable outcomes. The majority (10 out of 18) of those with favorable outcome tend to have baseline values of MDI larger than the mean (76.183 ms) and large normalized decreases over the 23 h period (13 out of 18), while the majority of patients with unfavorable outcomes (10 out of 13) had baseline MDI less than the mean and their MDI tended to increase (11 out of 13) over the 23 h period. **b** Scatter plot of baseline global longitudinal strain (GLS) vs. z-score normalized change in GLS (Absolute). Although the separation is not as pronounced as that of **a**, patients with favorable outcome (11 out of 18) tend to have a baseline GLS higher than the mean (− 6.51%) and a decrease (11 out of 18) in GLS over the 23 h period. Patients with unfavorable outcomes had baseline GLS less than the mean (7 out of 13), and a majority (8 out of 13) had an increase in GLS over the 23 h period

Given the small sample size, we present individual patient data in Fig. 3a, b, plotting the normalized absolute change in GLS versus the baseline GLS in Fig. 3a and the normalized absolute in MDI versus baseline MDI in Fig. 3b.

From a technical standpoint, of the 62 echocardiograms that made up the final study cohort, 26 of them had all 18 segments with adequate endocardial border tracings (as determined by the internal quality control system in the AFI software package), 18 echocardiograms had 17 segments that were suitable, 13 had 16 suitable segments, and 5 of the echocardiograms had 15 adequate segments. The segments that were most commonly found to be poorly tracked by the AFI software were: the apical anterior segment (16 times), the apical lateral segment (15 times), the apical posterolateral segment (9 times), and the apical anteroseptal segment (8 times). None of the other segments were found to be inadequately tracked more than 3 times. The average frames per second (FPS) for all of the 62 study echocardiograms was 68.3 with a frame rate range of 52 to 86 FPS.

## Discussion

We present preliminary but highly promising data regarding the potential role of strain-based echocardiographic parameters in determining response to treatment and association with 30-day readmissions for patients hospitalized with AHF. To our knowledge, this is the first study of its kind prospectively evaluating MDI as a treatment response variable in AHF.

Our results are unique and informative, as they provide the first report of speckle-tracking-derived MDI data obtained from pre-treatment POC echocardiograms in patients with AHF. The change in MDI from the baseline echocardiogram to 23-h follow-up has never been reported in the published literature on AHF. Mechanical dispersion is an index of inter-segmental discoordination of contraction which evaluates the differences in time-to-peak shortening for each of the LV segments that are analyzed. A key strength of MDI is that it can be assessed using the same recording(s) as GLS and thus adds very little time to the interpretation.

Previous research has shown impaired GLS to be associated with poor outcomes in patients with HF, MDI, however, is far less studied in HF than GLS. In fact, until recently, nearly, all of the literature on MDI focused on its association with ventricular arrhythmias and its role in CRT [13, 17, 18]. A recent study by Chan et al. investigated both MDI and GLS as predictors of outcomes in hospitalized patients with HF with reduced ejection fraction (HFrEF), reporting that both GLS and MDI were independent predictors of long-term outcome after adjustment for baseline variables [16]. GLS > − 7.8% and MDI > 72 ms were predictive of all-cause mortality or need for heart transplantation. Limitations of this study include the inclusion only of HFrEF subjects as well as the timing of echocardiograms with a median interval between admission and echocardiography of 10 days, rendering this measure less meaningful to the physician at the POC. This study does, however, lend support to the notion that MDI is a predictor of post-discharge outcomes in hospitalized HF patients, results that are consistent with our findings.

A potential mechanistic explanation for the observed change in MDI during treatment is that in patients with AHF, LV filling pressure is invariably increased. This elevated intra-cavitary pressure has the greatest impact on the subendocardial fibers as a result of their location immediately adjacent to the LV cavity. These fibers are oriented in a longitudinal fashion, thereby leading to the longitudinal strain parameters (including GLS and MDI) being the most sensitive of all the various strain measures [19]. Time-to-peak systolic strain for a given segment of the LV (the standard deviation of which is MDI) may be impaired by either: (1) decreased blood flow through the compromised small circulatory beds in the subendocardium which is the terminus of epicardial-to-endocardial coronary blood flow (the classical subendocardial ischemia model) or (2) pressure-induced decline in conduction velocity in areas with a high density of conducting fibers (i.e., the interventricular septum) [20, 21]. We suspect that during AHF episodes, a combination of these factors causes derangement in LV function. Thus, treatments geared towards reducing LV pressure (i.e., afterload reduction through vasodilation and non-invasive positive pressure ventilation) may be the key to maximizing improvements in GLS and MDI during the first 23 h of AHF therapy. In the present study, 23-h change in MDI proved to be a superior predictor than 23-h change in GLS; one reason for this may lie in the contractile potential of cardiomyocytes in AHF patients. There are likely limitations to the degree to which deformation (GLS) can improve with optimal treatment and thus many patients in our study exhibited substantially improved coordination of contraction timing (MDI) but only experienced small improvements in GLS after 23 h.

Our study has several important limitations, including small sample size and non-consecutive sampling of patients based on study team availability. Despite the relatively long time frame for the project, a study team member was available to enroll eligible subjects on average 2 days a month thereby placing significant limitations on our ability to capture patients. As previously mentioned, 13 patients had to be excluded from the final data analysis because of technical issues related to image quality or ECG-tracing quality. The majority of the ECG exclusions occurred early on during the study as our team was gaining familiarity with the precise timing necessary to capture clips that had three consecutive sinus beats with a good ECG waveform. The small number of subjects included for the final analysis thus raises the possibility of selection biases. To evaluate for this bias, we present data in Table 1 comparing the data from our study population with that of an AHF registry study also being conducted at our institution. The similarity between these 2 cohorts suggests that we captured a cohort of patients that was similar to the general population of AHF patients admitted at our institution.

We enrolled a fairly homogenous group of AHF patients, primarily a hypertensive, exclusively African-American cohort, and did not specifically evaluate or differentiate between patients with HFpEF versus HFrEF. It is possible that inclusion of a more diverse group of patients would alter our results; future studies are needed to address these questions. There were also differences in rates of CAD between the favorable outcome and readmitted cohorts (50% vs. 76.9%). Although this difference did not achieve statistical significance, it is certainly quite likely that CAD may have been the primary factor differentiating the primary outcome and this study was not powered to detect this difference. Future investigations would be wise to explore the role of CAD in LV longitudinal strain and how it influences short-term outcomes. Owing to the observational nature of this study, there is potential confounding from differences in treatments between patients. Another result of the observational and non-interventional design of the study is that invasive hemodynamic data (such as central venous pressure, pulmonary capillary wedge pressure, and central venous oxygen saturation) was not available for any of the patients in the study cohort. Comparison between the non-invasively derived indices of contractility using speckle-tracking and these established invasive parameters would have allowed for further hypothesis generation about the nature of the changes in speckle-tracking parameters that the study patients underwent. Future studies should ideally include these comparative data as well as echocardiography data on diastolic parameters obtained at the same time as the speckle-tracking data. Finally, there is some element of selection bias owing to the inherent limitations of speckle-tracking techniques—patients with atrial dysrhythmias or heart rate greater than 120 cannot be included; these limitations should not, however, preclude its use when feasible.

## Conclusions

Based on the results of this study, the speckle-tracking-derived LV strain parameters MDI and GLS appear to undergo significant change during the treatment of AHF, and the magnitude of this change demonstrated an association with 30-day readmission rates. While our study is limited by a small sample size in a distinct patient population, data are promising, hypothesis generating only, and supportive of the need for further investigation of MDI and GLS as variables to help guide treatment and predict short-term outcome in AHF.
